# Pyosalpinges after hysterosalpingography in a patient with lower genital tract infection and managed by laparoscopic surgery in a resource low tertiary hospital case report and literature review

**DOI:** 10.1186/s40738-018-0047-3

**Published:** 2018-04-13

**Authors:** Thomas Obinchemti Egbe, Fidelia Mbi Kobenge, Metogo Mbengono Junette Arlette, Eugene Belley-Priso

**Affiliations:** 1 Faculty of Health Sciences, University of Buea and Department of Obstetrics and Gynecology, Douala General Hospital, P.O. Box 63, Buea, Cameroon; 2 Department of Obstetrics and Gynecology, Douala General Hospital, Douala, Cameroon; 3 Department of Anesthesiology and Reanimation, Douala General Hospital, Douala, Cameroon; 4 Faculty of Medicine and Biomedical Sciences, University of Yaounde 1 and Department of Obstetrics and Gynecology, Douala General Hospital, Douala, Cameroon

**Keywords:** Pyosalpinges, Pelvic inflammatory disease, Infertility, Laparoscopy, Ultrasonography, Magnetic resonance imaging

## Abstract

**Background:**

Pyosalpinges (a complication of pelvic inflammatory disease) is infection of the fallopian tubes and the morbidity associated with it has major health implications. We are reporting a case of pyosalpinges diagnosed after hysterosalpingography and managed by laparoscopic surgery at the Douala General Hospital, Cameroon.

**Case presentation:**

A 29-year-old single woman, an assistant nurse of the Douala tribe in Cameroon. She is G1P0010 and came to our attention because of secondary infertility of three years duration. She has a history consistent with four lifetime sexual partners, self-medication for chlamydia trachomatis infection and induced abortion by dilatation and aspiration. Furthermore, she is HIV positive and had an ultrasound scan suggestive of bilateral hydrosalpinges. After a hysterosalpingography examination she developed painless muco-purulent vaginal discharge and bilateral adnexal tenderness on bimanual examination suggestive of pyosalpinges. Vaginal and cervical cultures isolated Ureaplasma urealyticum and Gardnerella vaginalis sensitive to ofloxacin and metronidazole, respectively.

At laparoscopy, bilateral pyosalpinges, pelvic adhesions and peri-hepatic adhesions were found. Bilateral salpingectomy with adhesiolysis including lysis of perihepatic adhesions and peritoneal toileting was done. She was discharged from hospital 72 h later and her hospital stay was uneventful. She was counseled for in-vitro fertilization and to register in the national HIV treatment programme. Her husband was prescribed ofloxacin empirically.

**Conclusion:**

Antimicrobial prophylaxis should be given to patients prior to HSG, especially those with a history of chlamydia or evidence of hydrosalpinges. There should also be universal STI testing in high risk and HIV positive patients or the danger for suboptimal antibiotic usage in areas where self-medication is common.

In resource-low tertiary hospitals where computed tomography or magnetic resonance imaging is not readily available and/or affordable, clinical examination and pelvic ultrasound remains the key diagnostic tool. Surgical treatment is the best option for pyosalpinges and when plausible, laparoscopic surgery is the treatment of choice. Laparotomy is the mainstay in most hospitals in Cameroon. The parent of the patient did not consent to histo-pathologic examination.

**Electronic supplementary material:**

The online version of this article (10.1186/s40738-018-0047-3) contains supplementary material, which is available to authorized users.

## Background

Pyosalpinges (a complication of pelvic inflammatory disease [PID]) is the infection of both fallopian tubes and the morbidity associated with it has major health implications [1]. PID frequently develops among sexually active women of reproductive age (15 to 49 years), especially those with multiple sex partners although it has also been described among virgins [2, 3]. PID has a worldwide distribution, but its incidence is higher in low-income countries like Cameroon [4]. In recent years, the incidence of PID has increased in sub-Saharan Africa and this has essentially been attributed to the human immunodeficiency virus (HIV) infection [4].

Furthermore, the prevalence of N. gonorrhea has reduced considerably in recent years [4, 5], but other micro-organisms like Mycoplasma genitalium, bacterial vaginosis, and anaerobes have also been implicated with PID [6, 7].When the genital organs of young females is infected by N. gonorrhea, C. trachomatis or any of the other microbial agents, it causes irreversible damage to the fallopian tubes, resulting in ectopic pregnancy or infertility that may be difficult to treat both by medical and surgical methods [4, 8].

The clinical presentation of PID has classically been heralded by the abrupt onset of severe lower abdominal pain during or shortly after menstruation, although it is now well recognized that both the onset and severity of symptoms can be more ill-defined and subtle [4, 9].

Ascending infection from the vagina and cervix is often due to sexually acquired infections with Neisseria gonorrheae or Chlamydia trachomatis. Sexually transmitted Mycoplasma genitalium has been identified as a likely cause of cervicitis, endometritis, salpingitis and infertility [9]. Sexual intercourse and retrograde menstruation may be particularly important for the movement of organisms from the lower to the upper genital tract [4].

Anaerobic and facultative bacteria that are found in vagina flora have been isolated alone or with Neisseria gonorrheae or Chlamydia trachomatis infection in the fallopian tubes of women with pelvic inflammatory disease [10, 11]. Other factors associated with ascending infection are intrauterine device (IUD) [12] and induced abortions [13].

If PID is not well treated, it may lead to pyosalpinges [1] that may sometimes rupture accidentally leading to peritonitis [14, 15].

The diagnosis of pyosalpinges is by medical imaging. Transvaginal ultrasonography, computed tomography and magnetic resonance imaging (MRI) revealing thickened, fluid-filled tubes are available during the diagnostic work-up and are highly specific for salpingitis [16].

However, the sensitivity of ultrasonography is only fair, and although CT-scan and MRI have high sensitivity, they are expensive and not typically available in low-income countries like Cameroon.

The challenge in diagnosis remains in differentiating pyosalpinges from hydrosalpinges which refers to fluid collection in the tubes. A pathognomonic finding of hydrosalpinges at US, CT, and MR imaging is a “cogwheel” appearance of the tubes when imaged in cross section, an appearance that is due to thickened longitudinal folds [16].

Pyosalpinges may be treated using antibiotics to fight the infection. Persistent pyosalpinges or ruptured pyosalpinges require surgical treatment with removal of the infected tubes [8, 9].

We are presenting the case of a young woman who developed pyosalpinges after HSG that was managed by laparoscopic surgery at the Douala General Hospital, Cameroon.

## Case presentation

A 29-year-old single woman, an assistant nurse of the Douala tribe in Cameroon. She is G1P0010, and sexually active since the age of 18. She has been frequenting her present partner for the past three years and came to the outpatient unit of the Department of Obstetrics and Gynecology of the Douala General Hospital, Cameroon, with the complaint of inability to conceive for 3 years. She has a past history of self-medication for C. trachomatis infection and induced abortion (dilatation and aspiration) at 8 weeks gestation, five years and nine years earlier, respectively. She also has a history of four life-time sexual partners but denies use of any form of contraception. She is currently not experiencing any pelvic pain or dyspareunia.

On Physical examination, the conjunctivae were not pale, the breast and abdominal examination were unremarkable. There was no vaginal discharge on speculum examination but there was mild cervical motion tenderness on bimanual examination. Vaginal and cervical cultures for Chlamydia trachomatis, N. gonorrhea and bacterial vaginosis were negative. Syphilis serology was also negative.

**Fig. 1:**
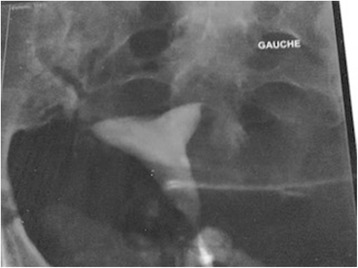
Hysterosalpingography showing normal uterine cavity and bilateral proximal tubal occlusion

A pelvic ultrasound scan showed a normal uterus measuring 76 × 47 mm with bilateral tubular structures with well-defined echogenic wall, a folded configuration, and linear echoes protruding into the tubal lumen suggestive of bilateral hydrosalpinges. Both ovaries were normal. A hysterosalpingography (HSG) done with antibiotic prophylaxis (amoxicilline-clavulanic acid) showed a normal uterine cavity with bilateral proximal tubal obstruction (Fig. 1). Her current partner denied follow-up.

**Fig. 2:**
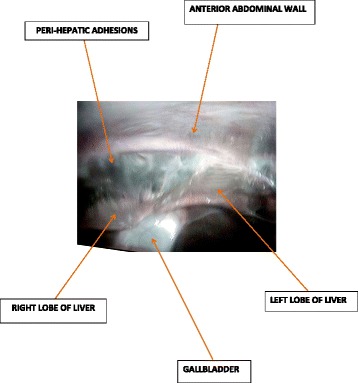
Showing peri-hepatic adhesions

**Fig. 3:**
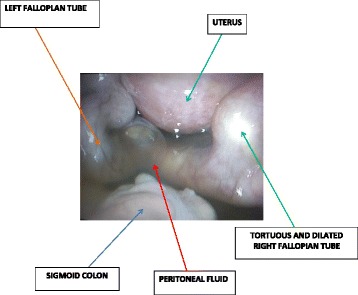
Panoramic view of pelvic organs

**Fig. 4:**
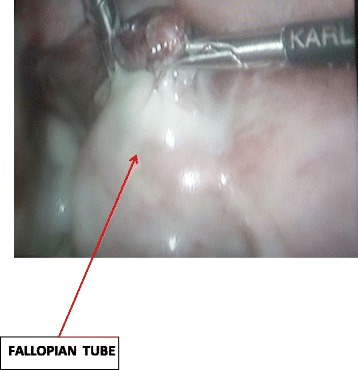
Drainage of pus from the fallopian tube

**Fig. 5:**
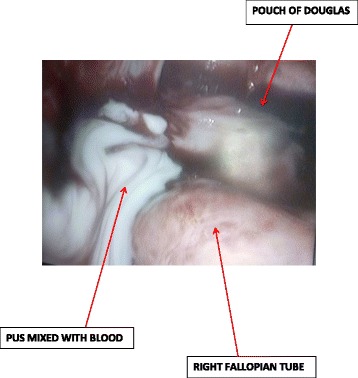
PUS mixed with blood in the pouch of douglas

She came back four days after the HSG with a painless moderate mucopurulent vaginal discharge requiring the use of panty liners. Her physical findings showed a normal temperature of 37.2 °C, blood pressure 120/80 mmHg and pulse 84 beats per minute. There was a non-tender depressible abdomen. On speculum examination, there was a creamy non-offensive (muco-purulent) vaginal discharge from the cervical os and bimanual examination revealed cervical motion tenderness and adnexal tenderness. Vaginal and cervical samples were taken for culture and sensitivity (gonorrhea, chlamydia trachomatis, Mycoplasma genitalium, Bacterial vaginosis and anaerobes). We also requested for a full blood count, coagulation studies, C-reactive protein, human immunodeficiency virus (HIV) serology, HBsAg and started antimicrobial therapy with ofloxacin; 400 mg bid, metronidazole; 500 mg tid and ketoprofene suppositories; 100 mg bid. We had a presumptive diagnosis of bilateral pyosalpinges so we counseled the patient for the possibility of bilateral salpingectomy with subsequent In-vitro fertilization and booked her for laparoscopic surgery. At laparoscopy, there were abundant peri-hepatic adhesions (perihepatitis or Fitz-Hugh-Curtis syndrome) (Fig. 2), free serous peritoneal fluid that was collected and sent for bacteriology and acid-fast bacilli. Both tubes were whitish in colour, swollen, severely inflamed and distorted with bilateral distal obstruction (Fig. 3). There were also intestino-uterine and tubo-ovarian adhesions. We did bilateral salpingostomy and drained abundant purulent secretions from both tubes (Figs. 4 and 5). The mucosa of both tubes was completely absent with very thick tubal wall (Tubal score 4). We carried out complete pelvic adhesiolysis, bilateral salpingectomy and lysis of the peri-hepatic adhesions (Additional file 1). Some of these adhesions were sent to the laboratory for C. trachomatis testing.

The family of the patient did not consent to histo-pathologic analysis of the removed tubes and preferred to carry out traditional rites which entailed among other things burying the tubes. The patient was discharged from hospital 72 h after surgery and her hospital stay was uneventful.

The results of laboratory work-up came out after surgery showing that the C-reactive protein was elevated 18 mg/l, white blood cell count was 12.000/mm3, polymorphonuclear neutrophils were 72%, HIV was positive, Ureaplasma urealyticulum was positive and Gardnerella vaginalis was present. These microorganisms were sensitive to ofloxacin and metronidazole so we continued the treatment we had started for 15 days and counseled the patient to register in the national HIV treatment programme. The husband was prescribed ofloxacin empirically since he was not compliant to follow-up.

## Discussion

The World Health Organization estimates 131 million new cases of C. trachomatis genital infection occur annually. Globally, infection is most prevalent in young women and men (14–25 years), likely driven by asymptomatic infection, inadequate partner treatment and delayed development of protective immunity [17]. Chlamydia trachomatis is the leading cause of bacterial sexually transmitted infection (STI) globally, especially in developing nations where routine laboratory diagnosis is unavailable [18]. However, in the index case we found instead U. urealyticum and G. vaginalis in the vaginal cultures. This is consistent with previous reports that PID could be associated with micro-organisms other than C. trachomatis or N. gonorrheae [6, 7]. Similarly, in a study carried out in San Francisco General Hospital, Pelvic inflammatory disease was shown to be a complex polymicrobial disease. The study demonstrated that risk factors associated with pelvic inflammatory disease cases could be differentiated by microbial etiology. Being of the black race was associated with STD pelvic inflammatory disease and recent IUD use was associated with non-STD pelvic inflammatory disease [19].

Furthermore, several risk factors have been identified on the index case which are associated with PID: Black race, early start of sexual intercourse (18 years old), multiple (four) sex partners, unprotected (no contraception) sexual intercourse, previous self-medication treatment for C. trachomatis infection, HIV positive and history of induced abortion by dilatation and aspiration. These factors have been consistently associated with causing ascending infection to the upper genital tract [20, 21]. It has been reported that being HIV positive increases the risk of PID [4]. Oral contraception has been reported to have a protective effect on PID [22]. However, the index case was not using any form of contraception. Symptoms of lower genital tract infection are often mild or absent but ascending infection in some women may lead to PID, including pelvic adhesions, tubal phimosis, tubal obstruction, hydrosalpinges, pyosalpinges and tubo-ovarian abscess. These result in reproductive sequelae such as ectopic pregnancy, infertility and chronic pelvic pain [8, 9]. Some studies have also reported PID among adolescent female virgins with and without tubo-ovarian abscess [3, 23]. Unfortunately, though pyosalpinges is common in sub-Saharan Africa, very few studies have been carried out on its prevalence. One study in Gabon reported the determinants of infertility among 340 women in eastern Gabon, an area situated in the “infertility belt” of Central Africa. Fallopian tube occlusion was diagnosed in 82.8% of cases, showing the importance of infection-related causes. Women with tubal occlusion did not differ significantly from women with normal tubes in obstetrical history or prevalence of Neisseria gonorrheae or Chlamydia trachomatis on endocervical culture. Antecedents of pelvic inflammatory disease or a pelvic mass were significantly more common in the group with tubal occlusion. This group also had a significantly higher prevalence of serum chlamydial antibodies at a titer of or higher [24]. The predominance of infection-related causes of infertility makes it imperative to focus resources on prevention programs of upper genital tract infections in women in low-income settings. The index case was diagnosed HIV positive at the same time as pyosalpinges were diagnosed. There are previous reports of the association of PID and HIV including trabecular pyosalpinx [25]. However, this patient was negative for Mycobacterium tuberculosis.

### Diagnosis of pyosalpinges

The goal standard for the diagnosis of PID has been by laparoscopy [4]. However, laparoscopy is an invasive procedure, costly for patients and in our setting offered only by few centres and surgeons. It therefore becomes difficult for laparoscopy to be accessible to a greater part of the population in Cameroon. The result is that we rely more on the clinical syndromic diagnosis of PID complemented by ultrasonography. Clinical assessment and the triad Ultrasonography, CT-scan and MRI with clinical pathological correlation have been recommended for diagnosis of tubal disease [16]. However, the diagnosis of PID can be challenging because the clinical manifestations may mimic those of other pelvic and abdominal processes. Given the nonspecific clinical manifestations, computed tomography (CT) is commonly the first imaging examination performed in high-income countries [16]. These examinations could enable us make the differential diagnosis with other pelvic conditions like endometriosis [26], appendicitis, ovarian malignancy [16] etc. However, CT-scan and MRI are not cost effective in low-income country like Cameroon.

One of the complications of PID is infertility due to tubal blockage, usually diagnosed by HSG. To avoid risks of pyosalpinges rupture [15], prior good quality imaging diagnosis to better appreciate the tubal structures should be done. The index case underwent a HSG and thereafter she developed symptoms of pyosalpinges. This procedure notwithstanding, the standard practice in our hospital is to perform vaginal cultures and sensitivity, then treat any underlying infection before performing HSG. We also administer prophylactic antibiotics on those with negative vaginal cultures prior to HSG. The dilemma in the index case is that the result of the HSG showed proximal tubal obstruction. However, it has been reported previously that laparoscopy performed after a two-sided abnormal HSG showed no abnormalities in 42% of the patients [27]. This implies that the tubes may have had instead distal tubal occlusion alone thereby explaining why the pyosalpinges manifested clinically after the HSG.

### Complications of pyosalpinges

PID has high morbidity; about 20% of affected women become infertile, 20% develop chronic pelvic pain, and 10% of those who conceive have an ectopic pregnancy. Repeated episodes of PID are associated with a fourfold to six-fold increase in the risk of permanent tubal damage [21]. Late complications of PID also include peritonitis caused by uterine and/or tubo-ovarian abscess rupture, development of peritoneal adhesions resulting in bowel obstruction and/or hydroureteronephrosis, right upper abdominal inflammation (Fitz-Hugh-Curtis syndrome), and septic thrombophlebitis [16].

This patient is an assistant nurse who was indulged in self-medication with probable sub-optimal doses that may explain why her infection remained silent and only flared-up after the HSG. The prevalence of self-medication was 70% (95%CI: 66.3–73.7) among 600 respondents in Ghana and the practice was significantly lower among medically inclined students (OR 0.2, 95% CI: 0.1–0.4, *p* < 0.001). Among the respondents who practiced self-medication in the study, the most common antibiotic used was amoxycillin (23.9%, 95% CI: 21.0–26.8%). Forty nine percent (95% CI: 44.2–53.8%) of the respondents had poor knowledge about the health implications of irrational use of antibiotics, and 46% (95% CI: 41.2–50.8%) did not comply with the completion of the full course of antibiotics [28].

Another study in India showed that 75% of pharmacy employees in the study were unlicensed practitioners, and the majority had very limited understanding of antibiotic resistance. Furthermore, only half could correctly define the term antibiotics. All reported that at times they dispensed antibiotics without a prescription. This practice was more common when treating patients who had limited access to a licensed physician because of economic or logistic reasons. Many pharmacy workers also felt pressure to provide shortened medication courses to poorer clientele, and often dispensed only 1 or 2 days’ worth of antibiotics. Such patients rarely returned to the pharmacy for the complete course [29]. However, the practice of self-medication is common in most low-income countries and is associated with disastrous health consequences [30, 31]. The main contributors to self medication could be related to lifestyle, socioeconomic factors, easy access to drugs, the increased potential and predilection to treat or manage certain illnesses through self-care, and greater availability of medicinal products.

Prior experience of self-medication and non-seriousness of the illness are the two important reasons for self-medication. The low severity of symptoms of illness has frequently been reported in the literature. Thus, the factors leading to self-treatment include age and gender, patient satisfaction with the healthcare provider, the price of the drugs, educational level, and socio-economic factors. Decreased healthcare budget may be an alarming reason in low-income countries. Interactions between prescribed drugs and the drugs taken for self-medication is an important risk factor of which healthcare providers must be aware of [30–33]. In Cameroon, the problem of self-medication has been compounded by the existence of road-side vendors who sell unprotected medication with doubtful expiry dates and sometime unsure of the active principle. Some of these medications may have just a placebo effect on the psychology of the patient thereby allowing the underlying medical condition to become more complicated.

Our patient had pelvic adhesions, peritoneal exudate, peri-hepatitis with grossly modified tubal morphology at laparoscopy. These were all signs of a longstanding infection. Therefore, because of the large burden of disease and risks associated with infection, several developed countries have instituted screening programs for C. trachomatis for women younger than 25 years old. This policy is expected to reduce the rates of PID by 50% after 5 years [34]. Unfortunately, we do not have such screening programs in Cameroon.

### Management of pyosalpinges

Quinolone regimens in which quinolones are used as a single agent or in combination with clindamycin produce high clinical cure rates, similar to those produced by a combination of a cephalosporin and doxycycline; (2) azithromycin regimens, often combined with nitroimidazole, produced high clinical cure rates; (3) newer data further demonstrate that the doxycycline-metronidazole combination produces unacceptable clinical cure rates, a finding previously reported [5], which is perhaps related to poor gastrointestinal tolerance; and (4) as expected, N. gonorrhoeae and, to a lesser extent, C. trachomatis were effectively eradicated with these newer regimens. Having been shown that quinolones and azithromycin are effective in producing acceptable rates of clinical cure of PID, it is noteworthy that previous reports indicate that N. gonorrhoeae is increasingly resistant to these agents and that another antibiotic should be counted on to eliminate N. gonorrhoeae [4, 8, 9, 35]. On the index case were isolated U. urealyticum and G. vaginalis sensitive to Ofloxacin and Metronidazole. We therefore used this medication for 15 days.

Transvaginal ultrasound-guided aspiration combined with antibiotics has been reported to be an effective and safe treatment regimen for tubo-ovarian abscess, with a high success rate of 93.4%. It has equally been recommended as a first-line procedure [36]. However, in our hospital we do not perform interventional radiography.

Laparoscopic surgery was performed on this patient where bilateral salpingectomy and adhesiolysis because she had bilateral pyosalpinges refractory to medical treatment. Laparoscopic salpingectomy was done by coagulating and dividing the proximal tube close to the cornua. The mesosalpinx was then serially coagulated and cut. It is prudent to stay close to the tube to avoid potentially compromising the ovarian blood supply that may jeorpardize ovarian function. Laparoscopic surgery has been practiced at the Douala General Hospital since 1994 and in certain health facilities in Yaounde, Cameroon [37, 38]. However, in most healthcare facilities in Cameroon, laparotomy is the mainstay of treatment for lack of expertise and equipment to practice laparoscopic surgery. Furthermore, the patient was also counseled to undergo In-vitro fertilization after laparoscopy [39]. Being young 29 years old might give a good yield of ovarian follicles for IVF and it has been reported previously that salpingectomy for hydrosalpinges improves IVF results [40, 41]. In Cameroon, IVF-ET is practiced in three centres; two private sector centres in Douala and one public IVF centre in Yaounde as reported previously [39]. The only difficulty she may face may be her family’s inability to handle the cost.

The parents of this patient did not consent to histopathology of the operative samples. This is consistent with the literature where in parts of Nigeria, people will hardly consent to a post-mortem examination [42]. Besides, it is customary in Cameroon that when a woman is not yet married as in the index case, the parents decide on her welfare.

## Conclusion

Antimicrobial prophylaxis should be given to patients prior to HSG especially those with a history of chlamydia or evidence of hydrosalpinges. There should also be universal STI testing in high risk and HIV positive patients or the danger for suboptimal antibiotic usage in areas/cultures where self-medication is common.

In resource-low tertiary hospitals where computed tomography is always not readily available and/or affordable clinical examination and pelvic ultrasound remains the key diagnostic tool. Surgical treatment is the best option for pyosalpinges and when plausible, laparoscopic surgery is the treatment of choice. Laparotomy is the mainstay of treatment for pyosalpinges in Cameroon. The parent of the patient did not consent to histopathologic examination.

We adhered to the principles of the Helsinki Declaration in the preparation of this case report.

## Additional file

Additional file 1: Laparoscopic salpingectomy. (3GP 13853 kb)

## Abbreviations

CT-scan: Computerized tomography scan
HIV: Human immunodeficiency virus
HSG: Hysterosalpingography
IUD: Intrauterine device
MRI: Magnetic resonance imaging
PID: Pelvic inflammatory disease
STD: Sexually transmitted disease
STI: Sexually transmitted infection
